# Molecular identification of *KRAS*, *BRAF*, and *PIK3CA* mutations in colorectal cancer patients from the Kurdistan region of Iraq

**DOI:** 10.22099/mbrc.2025.53059.2144

**Published:** 2025

**Authors:** Hamad Gihan Hamasharif

**Affiliations:** Department of General Science, College of Basic Education, Salahaddin University Erbil, Kurdistan Region of Iraq

**Keywords:** KRAS; BRAF; PIK3CA; CRC; Clinicopathological Features

## Abstract

Colorectal cancer (CRC) emerged due to genetic mutations that fuel tumor development and influence patient outcomes. This research investigates *KRAS*, *BRAF*, and *PIK3CA* mutations in Iraqi Kurdish patients to assess their biological relevance and impact on clinical outcomes. Clinical and pathological data were collected from 150 patients’ medical profiles. DNA was extracted from FFPE samples for *KRAS*, *BRAF*, and *PIK3CA* mutation analysis. Variations in *KRAS* and *BRAF* 600/601 were identified by polymerase chain reaction (PCR) amplification followed by hybridization assays. Real-time PCR was utilized to detect *PIK3CA* mutations. Tumors were predominantly located in the colon (80%) and classified as adenocarcinomas (88%), with stage III being the most frequent (36%). Metastases were observed in 72.67% of cases, primarily in the liver (46.67%). *KRAS* mutations were identified in 37.33% of cases (mainly in codons 12 and 13), while *BRAF* V600E mutations occurred in 10.67%, and *PIK3CA* mutations were detected in 18.67%, with exon 9 alterations more common than those in exon 20. *KRAS* mutations were strongly associated with liver metastases (*p*=0.006), and *BRAF* mutations correlated with peritoneal metastases (*p*=0.0001). Co-mutations of *KRAS* and *PIK3CA* appeared in 7.33% of cases, while *BRAF* and *PIK3CA* co-mutations were rarer (1.3%). Our study underscores the complexity of CRC and the pivotal role of *KRAS*, *BRAF*, and *PIK3CA* variations in tumor progression and outcomes in Iraq's Kurdistan Region, highlighting the importance of molecular profiling in clinical care.

## INTRODUCTION

Colorectal cancer (CRC) is a major contributor to global deaths, holding the third spot among cancers in men and the second among cancers in women. Even though there has been a significant enhancement in the management and therapeutic interventions for the disease, the fatality rate resulting from metastasis and recurrence post-resection remains considerably elevated [1]. The GLOBOCAN 2020 estimates reported 1.15 million new colon cancer, 0.7 million rectal cancer, and 50,000 anal cancer cases worldwide. By 2040, these numbers will rise to 1.92 million, 1.16 million, and 78,000, respectively [2]. Nevertheless, CRC's occurrence and fatality rates exhibit a varied distribution globally. In Iraqi Kurdistan, CRC is the third most common type of solid tumor, with reported incidence rates of 7.3% in males and 5.62% in females [3].

The heterogeneity of CRC develops through various variations in oncogenes, tumor suppressing, and genes associated with DNA repair. During this process, multiple cellular signaling pathways become activated and operate as key regulators of fundamental cell functions [4]. Among these signaling pathways is the epidermal growth factor (EGFR) signaling pathway, that significantly influences the formation of solid tumors. Nevertheless, *EGFR* mutations are relatively uncommon in CRC, occurring in only about 3% of cases [5]. Under normal conditions, *EGFR* activation triggers the RAS-RAF-MEK-ERK cascade, which supports cell growth and sustains survival. However, when RAS or RAF mutations are present, they lead to continuous activation of this pathway, fueling unchecked tumor growth and resistance to anti-EGFR therapies [6, 7]. The *KRAS* (Kirsten rat sarcoma virus) gene mutations are found in approximately 30–50% of CRC cases, enabling the pathway to remain active without requiring EGFR stimulation. Meanwhile, *BRAF* (B-Raf Proto-Oncogene, Serine/Threonine Kinase) gene mutation, principally the V600E variant, is detected in 8–15% of CRC patients and is correlated with more aggressive tumor progression and poorer survival outcomes [6, 8]. Since these genetic alterations render anti-EGFR monoclonal antibodies like cetuximab and panitumumab ineffective, alternative treatments, such as MEK inhibitors, are necessary for patients with *BRAF*-mutant tumors. As a result, molecular profiling has become an essential tool in personalized medicine, allowing for more precise patient stratification and the development of targeted treatment approaches [9, 10].

Most *KRAS* mutations (80%) occur in exon 2, essentially in codons 12 and 13, and impact anti-EGFR treatment responses. Less common mutations in exons 3 and 4 (codons 59, 61, 117, and 146) have been linked to a reduced sensitivity to anti-EGFR therapies [6]. Beyond their influence on treatment outcomes, *KRAS* mutations also serve as independent prognostic markers, each linked to distinct pathological characteristics [11]. The specific mutation site further influences tumor behavior. For instance, G12V mutations in codon 12 are often linked to more aggressive malignancies and poorer prognosis, whereas G13D mutations in codon 13 tend to respond better to targeted therapies [8]. Similarly, while *BRAF* V600E is the most prevalent *BRAF* mutation, other variants exist, leading to two distinct molecular subtypes of CRC: *BRAF* V600E-mutated and non-V600E-mutated. Each of these subtypes exhibits unique clinical and pathological features, further emphasizing the significant role played by molecular profiling in shaping treatment strategies [12].

The Phosphatidylinositol-4,5-Bisphosphate 3-Kinase Catalytic Subunit Alpha (*PIK3CA*) gene encodes the p110α catalytic subunit of PI3K, a principal player in the PI3K/AKT/mTOR signaling pathway, which regulates vital cellular activities such as proliferation, survival, metabolism, and angiogenesis [13]. This pathway is activated when receptor tyrosine kinases (RTKs) or G-protein-coupled receptors (GPCRs) trigger the phosphorylation of phosphatidyl-inositol 4,5-bisphosphate (PIP2) to phosphatidylinositol 3,4,5-trisphosphate (PIP3), leading to AKT activation. Consequently, AKT stimulates cell proliferation and enables cancer cells to resist programmed cell death by stimulating downstream effectors like mTOR [14]. Mutations in *PIK3CA*, especially in exons 9 and 20, result in continuous PI3K activation, driving uncontrolled tumor growth. These mutations are detected in around 10–20% of CRC cases, with those in exon 20 being associated with poor progression-free survival (PFS) and overall survival (OS) [15]. However, their prognostic impact is complex, as they frequently coexist with *KRAS*, *BRAF*, and *TP53* mutations. From a therapeutic viewpoint, *PIK3CA* mutations contribute to resistance against anti-EGFR therapies such as cetuximab and panitumumab. On the other hand, they may enhance sensitivity to mTOR and PI3K inhibitors, offering alternative treatment options [16]. Additionally, these mutations have been linked to increased PD-L1 expression and a higher tumor mutation burden (TMB), suggesting that tumors harboring them might respond more favorably to immune checkpoint inhibitors [15, 17].

Despite significant advancements in CRC treatment, distant metastasis remains the leading cause of mortality, intermittently driven by treatment resistance linked to genetic mutations in *KRAS*, *BRAF*, and *PIK3CA*, which alter disease progression [18]. Regardles of being studied extensively in other populations, the prevalence, distribution, and clinical impact of these mutations in the Kurdistan region remain underexplored. Given the influence of genetic, environmental, and ethnic factors on CRC pathogenesis, region-specific molecular studies are vital for enhancing treatment strategies and increasing patients' chances of survival.

## MATERIALS AND METHODS

### Patients:

This research, carried out in the Kurdistan Region of Northern Iraq from September 2022 to January 2025, involved 150 patients aged between 25 and 80 years, who were histologically confirmed to have CRC. Patients with Lynch syndrome, other cancers, or a history of ulcerative colitis were excluded. Clinical samples were collected from histopathology units in Erbil and Duhok, and patient records provided data on demographics and tumor characteristics. This research received ethical clearance from the Ethics Committee of Hawler Medical University, College of Medicine's Ethics Committee, referencing Meeting Code 7 and Paper Code 20. All patients provided written consent before taking part in this study, ensuring full compliance with ethical guidelines.

### Tumor DNA isolation:

 10-micrometer formalin-fixed, paraffin-embedded (FFPE) sections, ranging from 5 to 8 sections, were exploited for DNA isolation. Tissue sections were deparaffinized using xylene and ethanol standard protocol [19]. DNA was purified, following the manufacturer's instructions, using the Quick-DNA™ FFPE Kit (Zymo Research, Irvine, California, USA). The purity and concentration of DNA were determined with Implen NanoPhotometer N60 Micro-Volume UV-VIS Spectrophotometer (Implen GMBH, München, Germany).

### Detection of KRAS and BRAF mutations:

We utilized polymerase chain reaction (PCR) strip-based assay kits (ViennaLab Diagnostics GmbH, Vienna, Austria) to detect somatic mutations in *KRAS* and *BRAF*, following the manufacturer’s instructions. These kits are specifically designed to identify mutations that play a crucial role in prognosis and treatment decisions. The *KRAS* assay covers 29 mutations across several codons (12, 13, 59, 60, 61, 117, and 146), while the *BRAF* assay detects 9 mutations within codons 600 and 601. 

To amplify the DNA, we prepared samples with a concentration of 1–10 ng/µL and used 5 µL of DNA per PCR reaction on a SimpliAmp thermal cycler (Thermo Fisher Scientific, USA). The process started with an initial incubation at 37°C for 10 minutes, followed by 2 minutes at 94°C. Then, we ran 35 cycles with specific temperature settings: 94°C for 1 minute, 70°C for 50 seconds, 56°C for 50 seconds, and 60°C for 1 minute, ending with a final extension at 60°C for 3 minutes. Once the PCR was complete, we conducted a reverse hybridization step using nitrocellulose strips embedded with probes designed to recognize the target mutations. The strips were carefully washed, blocked, and developed according to the kit’s protocol, and the results were interpreted based on the distinctive color patterns that emerged. 

For quality control measures, these kits include a control line to validate the staining procedure, a PCR Positive Control to confirm the presence and quality of the PCR components and DNA template, and a PCR Negative Control to ensure specific amplification.

### Detection of PIK3CA mutation:

 The AmoyDex *PIK3CA* mutation detection kit (Amoy Diagnostics Co., Ltd., China) was used to identify mutations in the *PIK3CA* gene. The Kit is a real-time PCR test for in vitro diagnostics (IVD) that enables the qualitative identification of up to 11 somatic mutations in the *PIK3CA* gene including; exon 9: p.E542K, p.E545A, p.E545D, p.E545G, p.E545K, p.C420R, p.Q546E, and p.Q546R; and exon 20: p.H1047L, p.H1047R, and p.H1047Y (20). Before preparation of the PCR reaction mix, the sample DNA concentration was adjusted to a suitable concentration (10 ng/ml) as outlined in the manufacturer's instructions. By amplifying a housekeeping gene and utilizing the HEX channel included with the kit, the quality of the extracted DNA was assessed. A total of 47 cycles of amplifications were carried out: 95°C for 5 minutes, 1 cycle; 95°C for 25 seconds, 64°C for 20 seconds, and 72°C for 20 seconds, 15 cycles; and 93°C for 25 seconds, 60°C for 35 seconds, and 72°C for 20 seconds, 31 cycles. In the third stage, HEX and FAM signals were gathered. As outlined in the manufacturer's handbook, run files were examined and evaluated. 

For the quality control measures, the kit includes internal and positive controls, while we added no-template controls (NTC) to monitor amplification performance and exclude contamination. These measures were routinely applied during analysis to ensure assay accuracy, following the manufacturers’ instructions. 

### Statistical Analysis:

 To analyze the data, GraphPad Prism software (version 9.3.1, Boston, USA) was used. The occurrence of specific variants was correlated to the patient’s clinicopathological parameters using Fisher's exact and chi-square tests. Summarized data were obtained using descriptive statistics. For continuous variables, like age, data were presented as mean ± standard deviation. Differences between variables resulted in a *p*-value≤0.05, considered statistically significant.

## RESULTS

The study examined 150 FFPE specimens of CRC, revealing key demographic and pathological characteristics. The mean age of patients was 65.5±5.2 years, with an age range of 25 to 85 years. The majority of cases (74.67%) were individuals aged over 60 years. Gender distribution showed a higher prevalence in males (62.67%) compared to females (37.33%). 

The colon was the most frequent tumor site, accounting for 80% of cases, while 20% of tumors were located in the rectum. Adenocarcinoma was the predominant tumor type, comprising 88% of cases, while other histological subtypes made up the remaining 12%. Regarding tumor differentiation, 58.7% of cases were moderately differentiated, 28% were well-differentiated, and 13.3% were poorly differentiated. The distribution of tumor grades showed that most cases (70.67%) were classified as G2, while 20% fell into G3, and only 9.33% were in the G1 category. Tumor staging analysis indicated that stage III was the most common, representing 36% of cases, followed by stage II (29.34%), stage IV (25.33%), and stage I (9.33%). The metastatic spread was observed in 72.67% of cases, with the liver being the most frequently affected site (46.67%), followed by the lungs (15.33%), and the peritoneum (10.67%). A smaller proportion (27.33%) exhibited no metastasis.

*KRAS* variations were detected in 37.33% of cases (56/150) and *BRAF* mutation in 10.67% of cases (16/150). Collectively, mutations in both genes were identified in 48% (72/150) patients, and 52% (78/150) patients had none of these mutations. Among the *KRAS* mutations, the most common was G12D (c.35G>A) in codon 12, detected in 22 cases (14.67%). This was followed by G13D (c.38G>A) in codon 13, found in 14 cases (9.33%). Other *KRAS *mutations included G12V (c.35G>T) (5.33%), G13C (c.37G>T) (2.67%), Q61L (c.182A>T) (2.67%), and A146P (c.436G>C) (2.67%). The *BRAF* mutation V600E (c.1799T>A) was detected in 16 cases, corresponding to 10.67% of total CRCs.

*PIK3CA* gene mutations were identified in exons 9 and 20 of the gene. In exon 9, the most frequently observed mutation was E542K (c.1624G>A), detected in 11 cases (7.33%). This was followed by E545K (c.1633G>A) in 7 cases (4.67%) and Q546K (c.1636C>A) in 2 cases (1.33%). In exon 20, the H1047R (c.3140A>G) mutation was present in 7 cases (4.67%), while the H1047L (c.3140A>T) mutation was observed in only 1 case (0.67%).

The genetic landscape of CRC in our cohort of 150 CRC cases revealed definite links between *KRAS* and *BRAF* mutations and various clinical parameters (Table 1). *KRAS* mutations were observed in 37.3% of patients, while *BRAF* mutations were identified in 10.7%. Gender differences were evident in *BRAF* mutations, with females exhibiting a significantly higher mutation frequency (*p*=0.002). However, *KRAS* mutations showed no significant gender bias. Age did not play a significant role in the prevalence of either *KRAS* or *BRAF* mutations. The pattern of *KRAS* and *BRAF* mutations across different tumor sites (colon vs. rectum) was significantly associated with *KRAS* mutation (*p*=0.03). Oppositely, no site-specific preference was observed with *BRAF* mutation. Adenocarcinoma and other histological tumor types did not show significant differences in *KRAS* or *BRAF* mutation rates. Tumor differentiation was significantly associated with both mutations. Poorly differentiated tumors were more likely to harbor *KRAS* (*p*=0.04) and *BRAF* (*p*=0.004) mutations. Tumor grade followed a similar trend, with G3 tumors having a significantly higher prevalence of *KRAS* (*p*=0.01) and *BRAF* (*p*=0.0003) mutations. *KRAS* mutations showed a strong correlation with advanced tumor stages (*p*=0.04), particularly in stage IV tumors. However, *BRAF* mutations did not show a similar correlation with tumor stage. Metastasis patterns provided additional insights: *KRAS* mutations were strongly correlated with liver metastases (*p*=0.006), while *BRAF* mutations were more commonly linked to peritoneal metastases (*p*=0.0001). Lung metastases were observed more frequently in *KRAS*-mutated tumors.

**Table 1:** Association between mutations in *KRAS* and *BRAF* and clinicopathological aspects of patients

**Characteristics**	**n (%)**			***KRAS***					***BRAF***			
				**Wild ** **n (%)**	**Mutated n (%)**		***p***		**Wild ** **n (%)**	**Mutated n (%)**	***p***
**Gender**						0.68					0.002	
Male	94 (62.7)			56 (37.4)	38 (25.3)				90 (60)	4 (2.7)		
Female	56 (37.4)			38 (25.4)	18 (12)				44 (29.3)	12 (8)		
		
**Age Groups**						0.98					0.76	
≤ 60	38 (25.3)			24 (16)	14 (9.3)				35 (23.3)	3 (2)		
> 60	112 (74.7)			70 (46.7)	42 (28)				99 (66)	13 (8.7)		
**Tumor Site**						0.03					0.53	
Colon	120 (80)			70 (46.7)	50 (33.3)				108 (72)	12 (8)		
Rectum	30 (20)			24 (16)	6 (4)				26 (17.3)	4 (2.7)		
**Tumor Type**						0.8					0.1	
Adenocarcinoma	132 (88)			82 (54.7)	50 (33.3)				120 (80)	12 (8)		
Others	18 (12)			12 (8)	6 (4)				14 (9.3)	4 (2.7)		
**Differentiation**						0.04					0.004	
Poor	20 (13.3)			4 (2.7)	12 (8)				12 (8)	8 (5.3)		
Moderate	88 (58.7)			46 (30.7)	42 (28)				84 (56)	4 (2.7)		
Well	42 (28)			26 (17.3)	16 (10.7)				38 (25.3)	4 (2.7)		
**Tumor Grade**						0.01					0.0003	
G1	14 (9.3)			12 (8)	2 (1.3)				14 (9.3)	0		
G2	106 (70.7)			58 (38.7)	48 (32)				100 (66.7)	6 (4)		
G3	30 (20)			12 (8)	18 (12)				20 (13.3)	10 (6.7)		
**Tumor Stage**						0.04					0.3	
I	14 (9.3)			9 (6)	5 (3.3)				14 (9.3)	0		
II	44 (29.3)			28 (18.7)	16 (10.7)				40 (26.7)	4 (2.7)		
III	54 (36)			40 (26.7)	14 (9.3)				49 (32.7)	5 (3.3)		
IV	38 (25.4)			17 (11.4)	21 (14)				31 (20.7)	7 (4.7)		
**Metastasis Location **						0.006					0.0001	
Lung	23 (15.3)			17 (11.3)	6 (4)				21 (14)	2 (1.3)		
Liver	70 (46.7)			25 (16.7)	45 (30)				66 (44)	4 (2.7)		
Peritoneum	16 (10.7)			12 (8)	4 (2.7)				6 (4)	10 (6.7)		
No metastasis	41 (27.3)			41 (27.3)	0 (0)				41 (27.3)	0		

The coexistence of *PIK3CA* mutations with *KRAS* and *BRAF* mutations in CRCs was analyzed and summarized in Table 2. Among the *PIK3CA* mutations, exon 9 mutations (E542K, E545K, Q546K) were observed in 20 cases (13.33%), while exon 20 mutations (H1047R, H1047L) were detected in 8 cases (5.34%). *KRAS* mutations were predominantly found in exon 2, affecting codons 12 and 13 (G12D, G12S, G13D, G13C) in 48 cases (32%). Additionally, mutations in exon 3 (Q61L) and exon 4 (A146P) were detected in 4 cases each (2.67%). *BRAF* mutations were identified at exon 15, specifically the V600E variant, in 16 cases (10.67%). Co-mutation analysis revealed that *PIK3CA* and *KRAS* mutations coexisted in 11 cases (7.33%), whereas *PIK3CA* and *BRAF* co-mutations were found in only 2 cases (1.3%). Notably, *KRAS* and *BRAF* mutations were identified together in 1 case (0.67%), but no tumors exhibited concurrent mutations in *PIK3CA*, *KRAS*, and *BRAF*.

**Table 2:** Coexistence of *PIK3CA* mutations with *KRAS* and *BRAF* mutations in colorectal cancer

**Gene**	**Exon**	**Codon**	**Mutation subtypes**	**n (%)**
***PIK3CA***	9	542, 545, 546	E542K, E545K, Q546K	20 (13.33)
	20	1047	H1047R, H1047L	8 (5.34)
***KRAS***	2	12, 13	G12D, G12S, G13D, G13C,	48 (32)
	3	59, 61	Q61L	4 (2.67)
	4	146	A146P	4 (2.67)
***BRAF***	15	600	V600E	16 (10.67)
***PIK3CA*** **+** ***KRAS***	All	All	E545K, G12S, G12D, G13D, H1047R, E542K, Q546K	11 (7.33)
***PIK3CA*** **+** ***BRAF***	All	All	E542K, V600E, H1047R	2 (1.3)
***KRAS*** **+** ***BRAF***	2, 15	13, 600	G13D, V600E	1 (0.67)
***PIK3CA*** **+** ***KRAS*** **+** ***BRAF***	None	None	None	0

## DISCUSSION

Over the past decades, compelling amelioration in CRC management has been gained from a deeper understanding of its molecular mechanisms. However, the disease remains highly complex, with variable treatment responses and prognostic outcomes [21]. Although EGFR is a key therapeutic target in CRC, resistance to EGFR inhibitors remains a well-documented challenge. This resistance is often driven by *KRAS* mutations and influenced by factors such as altered ligand expression, elevated *EGFR* gene copy number, mutations in *BRAF* and *PIK3CA*, and activation of alternative signaling pathways [22]. Variants in *KRAS* (exons 2, 3, and 4), *BRAF* (exon 15), and *PIK3CA* (exons 9 and 20) are particularly significant as predictors of anti-EGFR therapy response [23]. This study examined these genetic alterations in 150 CRC patients to generate clinically relevant data, aiming to refine treatment strategies and improve patient outcomes.

The overall *KRAS* mutation rate was 37.4% in the present study. This prevalence is lower than previously reported rates in Iraq (48%), Western Europe (44.7%), and Indonesia (41%) (24-26). However, it closely aligns with mutation rates observed in Eastern Europe (35.8%) and China (36.1%) (25, 27). Interestingly, the Kurdistan region follows quite a bit similar genetic mutation patterns to neighboring countries. *KRAS* mutations are found in 37.4% of cases, aligning with rates in Iran (41%) (28) and Turkey (33.2%) [29]. The similarity points to both genetic and environmental factors at play, and it also underlines the significant differences in how common *KRAS* mutations are across various ethnic and geographic groups. This pattern suggests that inherited traits and local environmental conditions likely shape the specific mutation trends seen in Northern Iraq. 

In our study, *KRAS* mutations were mostly detected in codons 12 and 13 of exon 2, with a mutation frequency of 32% (48/150). In contrast, mutations in codons 61 and 146 of exons 3 and 4 were significantly rarer, each occurring at a frequency of 2.67%, aligning with prior research [30]. Whilst some studies suggest that *KRAS* mutations are more common in female patients and those with right-sided colon cancer [31], our data contradict this, showing a higher prevalence in males. Additionally, previous research has linked *KRAS* mutations to poor differentiation and increased metastatic lymph node count [32]. However, our findings differ, indicating an association with well- or moderately differentiated tumors, consistent with observations reported by Li et al. [30]. The complexity of *KRAS* mutation patterns in CRC is further underscored by studies investigating their correlations with patient age and tumor location [33].

The *BRAF* gene, a main proto-oncogene linked to cancer development and progression, exhibited a mutation rate of 10.67% (16/150 cases) in the present study, aligning with previous findings [12]. The prevalence in the current study is higher than reported rates in Iran (5.96%) (28) and Turkey (5.3%) (34). While still within global norms (5–15%), this increased frequency in the Kurdish population may be influenced by unique tumor biology or environmental factors. *BRAF* mutations were significantly associated (*p*<0.05) with factors such as female gender, poor tumor differentiation, high-grade malignancies, and peritoneal metastasis. These results are consistent with earlier research linking BRAF mutations to right-sided tumor localization, poor differentiation, and peritoneal spread [35]. Notably, 75% of *BRAF*-mutated tumors were located in the colon, reinforcing prior studies that associate the V600E mutation with colon tumors rather than rectal ones [36]. These findings underscore the crucial role of tumor location in shaping personalized treatment strategies for CRC, given the biological and clinical differences between colon and rectal cancers. 

The tumor stage plays a primary role in prognosis, particularly in cases involving *BRAF* mutations. A meta-analysis of seven phase III clinical trials demonstrated that *BRAF*-mutated tumors in stages II and III were significantly associated with poor outcomes [37]. Also, a study analyzed patients across stages I–IV indicated that while the V600E mutation was a strong predictor of a worse prognosis, other mutation subtypes in the same gene had no significant impact on patient survival [27]. These findings underscore the necessity of tailored treatment approaches based on tumor genetics alongside stage.

The present study uncovered a rare co-occurrence of *KRAS* (G13D) and *BRAF* (V600E) alterations in CRC, highlighting tumor heterogeneity and challenging the assumption that these mutations are mutually exclusive within the studied ethnicity. This aligns with findings from Deshwar et al., who identified such mutations in only 0.5% (4 out of 820) of CRC cases with liver metastases [38]. Among these cases, case 1 (*KRAS* G13D alongside *BRAF* V600E), case 2 (*KRAS* G12V plus *BRAF* V600E), and case 3 (*KRAS* G13D beside *BRAF* D594N) did not survive the disease within 485, 236, and 79 days, respectively, following liver excision. Case 4, with a T4 primary tumor and mutations in *KRAS* G12S and *BRAF* G469S, survived 416 days after surgery and received postoperative FOLFOX chemotherapy. In contrast, patients 1 and 2 underwent preoperative FOLFOX treatment. These variations emphasize the profound influence of specific mutation combinations and the timing of chemotherapy on patient survival.

*PIK3CA* is a commonly altered proto-oncogenes in CRC, with a 10–20% occurrence rate. It is a key regulator of cell growth and survival through the PI3K-AKT-mTOR pathway. When mutated, *PIK3CA* can drive tumor progression, increase invasiveness, and contribute to treatment resistance [13, 14]. In our study, 18.67% of CRC cases exhibited *PIK3CA* mutations, a rate that aligns with global trends (10–20%) and closely correspondence those found in Australia, France, Italy, and Japan (14%–17.8%) (39). However, this frequency was lower than southern Italy’s 28% but higher than rates reported in China, Poland, Singapore, Switzerland, and previous studies from India (2.2%–10.1%) [40]. Notably, the prevalence of *PIK3CA* mutations in the Kurdish population is slightly higher than Iran's reported rate of 13.24% (28) and Arab countries of 13.1% [41]. However, values fall within the globally observed range, indicating the consistent role of the PI3K/AKT signaling pathway in CRC development across different populations. The slightly higher prevalence of *PIK3CA* mutations in the Kurdish population may be shaped by unique genetic backgrounds or environmental influences, emphasizing the need to consider *PIK3CA* status when tailoring treatment plans to achieve better patient outcomes.

Mutation frequencies vary across different cancers, but in CRC, *PIK3CA* alterations are particularly notable due to their involvement in metabolic reprogramming and chemoresistance [42]. The prognostic impact of these mutations remains controversial, some evidence links them to shorter PFS and OS. In stages I–III CRC, they are generally associated with worse outcomes, with mutations primarily occurring in exons 9 and 20 [43]. This study found 13.33% variations in exon 9 and 5.34% in exon 20, which is consistent with previous reports but with some regional variability [44]. These differences may stem from population demographics, tumor heterogeneity, or differences in detection techniques. Given their significant role in tumor progression and therapy resistance, further research is essential to clarify their prognostic value and therapeutic potential, particularly in Kurdish CRC patients, where deeper insights could pave the way for more personalized treatment strategies.

E542K, E545K, and Q546K mutations of *PIK3CA* exon 9 emerged as the most frequent, representing 70% (20 out of 28) of detected cases. These mutations often co-occurred with *KRAS* or *BRAF* alterations, suggesting synergistic or interactive roles in CRC progression. The functional differences in *PIK3CA* mutations provide insight into this phenomenon: exon 9 mutations (E542K and E545K) require RAS-GTP binding to enhance *PIK3CA* activity, while exon 20 mutations (e.g., H1047R) operate independently of RAS-GTP signaling [45]. Specifically, *KRAS* and *PIK3CA* co-mutations were detected in 7.33% of cases, while *BRAF* and *PIK3CA* co-mutations were observed at a lower frequency of 1.3%. 

Studies suggest that *PIK3CA* mutations, when present alongside *KRAS* or *BRAF*, may reduce responsiveness to anti-EGFR therapies, thereby limiting treatment options. Additionally, co-mutations have been associated with increased tumor aggressiveness and poorer prognosis, emphasizing the need for alternative targeted treatment strategies, such as PI3K or MEK inhibitors in combination therapies [46]. The low occurrence rate of *BRAF* and *PIK3CA* co-mutations suggests these alterations may define distinct molecular subgroups within CRC. Furthermore, the absence of triple-mutant cases (*PIK3CA*+*KRAS*+*BRAF*) supports the hypothesis that *KRAS* and *BRAF* mutations represent alternative oncogenic pathways that rarely coexist with *PIK3CA* mutations. 

Like any research, this study has its limitations. The small sample size, in particular, may limit how broadly the findings can be applied, and the lack of treatment response and long-term outcome data. Future research employing advanced methods like next-generation sequencing is advised for a better understanding of the role of *PIK3CA*, *KRAS*, and *BRAF* alterations in Kurdish CRC patients and their impact on prognosis and therapy.

This study highlights the distinct mutational landscape of *KRAS*, *BRAF*, and *PIK3CA* mutations in CRC patients from the Kurdistan Region of Iraq. The low co-occurrence of mutations of *KRAS* or *BRAF* with *PIK3CA* in our cohort suggests unique oncogenic pathways that may be affected by racial and geographical distribution and underscore the heterogeneity of CRC at both clinical and molecular levels. The significant associations between *KRAS* and *BRAF* mutations with tumor differentiation, grade, and metastatic patterns highlight their prognostic relevance. Additionally, the presence of *PIK3CA* mutations suggests potential responsiveness to PI3K pathway inhibitors. 

Understanding the biological implications of genetic mutations, not just how often they occur, plays a vital role in shaping better treatment strategies for CRC. A computational research, has shown how certain mutations can influence cellular behavior in meaningful ways [47]. Building on these methods to explore gene mutations like *KRAS*, *BRAF*, and *PIK3CA* could provide deeper insight into how they function specifically within the Kurdistan population. By weaving molecular data into predictive modeling, there’s real potential to fine-tune targeted therapies and make patient care more precise and effective.
